# Cold Atmospheric Plasma: A New Strategy Based Primarily on Oxidative Stress for Osteosarcoma Therapy

**DOI:** 10.3390/jcm10040893

**Published:** 2021-02-23

**Authors:** Miguel Mateu-Sanz, Juan Tornín, Maria-Pau Ginebra, Cristina Canal

**Affiliations:** 1 Biomaterials, Biomechanics and Tissue Engineering Group, Department of Materials Science and Engineering, Escola d’Enginyeria Barcelona Est (EEBE), Universitat Politècnica de Catalunya (UPC), 08930 Barcelona, Spain; miguel.mateu@upc.edu (M.M.-S.); juan.tornin@upc.edu (J.T.); maria.pau.ginebra@upc.edu (M.-P.G.); 2 Barcelona Research Center in Multiscale Science and Engineering, UPC, 08930 Barcelona, Spain; 3 Research Centre for Biomedical Engineering (CREB), UPC, 08034 Barcelona, Spain; 4 Institute for Bioengineering of Catalonia (IBEC), Barcelona Institute of Science and Technology (BIST), 08034 Barcelona, Spain

**Keywords:** osteosarcoma, cold atmospheric plasma, plasma treated liquids, reactive oxygen and nitrogen species, oxidative stress, tumor microenvironment, cancer stem cells

## Abstract

Osteosarcoma is the most common primary bone tumor, and its first line of treatment presents a high failure rate. The 5-year survival for children and teenagers with osteosarcoma is 70% (if diagnosed before it has metastasized) or 20% (if spread at the time of diagnosis), stressing the need for novel therapies. Recently, cold atmospheric plasmas (ionized gases consisting of UV–Vis radiation, electromagnetic fields and a great variety of reactive species) and plasma-treated liquids have been shown to have the potential to selectively eliminate cancer cells in different tumors through an oxidative stress-dependent mechanism. In this work, we review the current state of the art in cold plasma therapy for osteosarcoma. Specifically, we emphasize the mechanisms unveiled thus far regarding the action of plasmas on osteosarcoma. Finally, we review current and potential future approaches, emphasizing the most critical challenges for the development of osteosarcoma therapies based on this emerging technique.

## 1. Background

Osteosarcoma (OS) is the most common type of primary solid tumor originating in bone. It predominantly affects children and young adults, whose bone cells are experiencing rapid growth and higher risk of mutation, as well as adults with bone pathologies [1]. OS is usually localized in the metaphysis of long bones, particularly the distal femur (30%), proximal tibia (15%) and humerus (15%) [2]. Approximately 20% of patients present with metastases at early stages [3], with the lung being the most common site of metastasis and distal bones being the second most common [4], and usually present with a high grade of metastasis in the long term. Although OS occurs with a low incidence compared with other solid tumors, it is ranked among the most frequent cause of cancer death in childhood, with a 5-year survival of less than 30% in metastatic patients [5] and 20% for recurrent tumors [6].

Today, surgery combined with chemotherapy is the first-line treatment [3,7], but it lacks complete effectiveness and is associated with harmful effects [8]. Therefore, the development of novel therapies is required to improve the outcomes of OS patients. In the last several years, a new anti-cancer therapy based on the application of Cold Atmospheric Plasma (CAP) has shown promising results in a wide range of tumor types (pancreatic, cutaneous, lung and colon carcinomas, neuroblastoma, lymphoma, etc.) in in vitro studies, twenty-seven in vivo studies [9] and three clinical trials, and studies are ongoing [10].

In the last few years, there has been a significant increase in the number of studies reporting the in vitro efficiency of CAP in OS. In this context, the aim of this review is to comprehensively compile the existing literature in the field and to discuss whether CAP application could be considered to treat this disease. For this purpose, we consider the current challenges in proposing an effective therapy for OS, highlighting its possible impact along with the different malignant features of this kind of cancer, and we discuss future developments that will allow this therapy to advance.

## 2. Current Treatment in OS

The prevalent procedure for the treatment of OS is surgical resection, followed by intravenous chemotherapy (mainly doxorubicin, cisplatin and methotrexate). In cases in which surgery is not sufficient, radiotherapy is necessary to complete local treatment [7]. This strategy has led to a significant increase in 5-year survival rates, but survival rates for metastatic and recurrent tumors are still very low. Furthermore, these therapies are highly invasive, produce side effects and do not ensure complete eradication [8,11], emphasizing the need for a better understanding of the current state of OS treatment to advance the development of novel therapies.

### 2.1. Surgery

Complete surgical resection of the malignant tissue is the first option for OS treatment (Table 1), which is critical to obtain remission and improve patient survival. For effective surgical resection, OS tumors should be removed with accurate margins to prevent residual disease and tumor recurrence. When tumor resection cannot be achieved without generating a non-functional limb, limb amputation must be considered. On the other hand, for cases in which OS tumors can be resected while preserving limb function, preservation surgery is employed [12]. In most cases, limb preservation surgery can be complex, and bone reconstruction strategies are required [13].

Limb preservation surgery poses an increased risk of non-visible tumor remnants in the case of localized tumors, and complete tumor eradication may not be feasible in metastatic OS [8]. For these reasons, most OS patients receive neoadjuvant chemotherapy before surgical resection to decrease tumor size and achieve adequate margins in addition to receiving it postoperatively to eradicate tumor remnants and avoid metastasis [4]. In addition to surgery, radiotherapy can be also used to add local control and may improve patient survival [8].

### 2.2. Chemotherapy

The most commonly used chemotherapeutic regimen for OS includes the systemic administration of a three-drug combination of methotrexate, doxorubicin and cisplatin (Table 1) [14,15]. Methotrexate acts as an antimetabolite that interferes with the metabolism of folic acid, which is essential to produce DNA, thus interfering with cell division [16]. On the other hand, doxorubicin and cisplatin constitute DNA-intercalating agents and thus interfere with cell division. Doxorubicin inhibits the progression of the enzyme topoisomerase II, which relaxes supercoils in DNA for transcription, thereby stopping the process of replication [17], whereas cisplatin cross-links purine residues, producing DNA adducts [18]. Data also suggest that doxorubicin and cisplatin produce free radicals that induce DNA and cell membrane damage and interfere with mitochondrial respiration [17,18,19].

The combination of surgery and chemotherapy has increased the 5-year survival for patients with non-metastatic OS from 25 to 70% [14]. However, this survival rate has plateaued for patients with non-metastatic disease, and the prognosis is only about 20% for patients with metastases [11]. Many patients develop chemoresistance, leading to tumor relapse and metastasis after treatment cessation. Additionally, side effects that produce crystal nephropathy and systemic oxidative stress, which leads to hepatotoxicity and cardiac damage [8,17,18,19], are associated with this therapy.

### 2.3. Radiotherapy

Radiotherapy damages the DNA of tumor tissues, leading to cell death. Ionizing radiation is also able to damage numerous cell organelles, primarily through the production of hydroxyl radicals (·OH) [20]. OS has a low response to radiotherapy, so it is not the first option for treatment and is only considered after chemotherapy in specific cases (Table 1) [21,22]. To elicit a therapeutic response, high doses of radiation are required, which increases adverse effects, such as its detrimental action on normal tissue, systemic cytotoxicity and the risk of radiation-induced secondary tumors [23,24,25].

### 2.4. Other Therapeutic Options

Due to the multiple drawbacks of the current strategies for OS, the development of innovative therapies is of great interest. The widest body of research is focused on either immunotherapy, i.e., the stimulation and/or use of components of the immune system to increase the immune response against cancer cells, or on targeted therapies based on the use of different kinds of inhibitors of critical proto-oncogenes (Table 1). In OS, different cytokines have been investigated as immunomodulatory agents [7]. For targeted therapies, several proto-oncogenes, such as protein kinases [26], have been proposed. Different inhibitory agents, such as small molecules [27,28] and siRNA [29,30], have been reported to silence these proto-oncogenes and proteins related to poor prognosis in OS.

Despite the great research efforts in these areas, the complex hallmarks that characterize OS make it difficult to develop effective therapies. Although the idea of stimulating the host immune system is highly attractive, OS possesses a remarkable ability to evade the immune system response. Moreover, immunotherapies can trigger autoimmune responses and chronic inflammation [31]. On the other hand, the increased heterogeneity and genetic instability of OS make it difficult to find effective targets for its treatment [32,33]. In this field of research, a rising new therapy based on CAP has shown great promise for cancer treatment, especially for OS. The advances made thus far are compiled in the following sections.

## 3. CAP for Cancer Therapy

**Figure 1:**
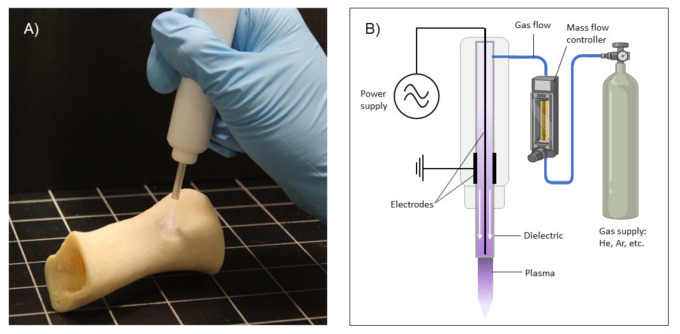
(**A**) Atmospheric pressure plasma jet (APPJ) in operation. (**B**) illustration of the principle of generation of plasma, where a power discharge is applied to two electrodes in between a gas (usually He or Ar) flows through a dielectric tube. This generates the plasma discharge that can then be applied directly to OS tumors in vivo, cells in vitro or used to produce plasma-treated liquids that can in turn be used to treat tumors or cells.

Plasma is a multi-component, chemically active and highly reactive state of matter [34] consisting of a totally or partially ionized gas that produces several uncharged and charged particles (i.e., ions, electrons, atoms, molecules and free radicals), electromagnetic fields and visible–ultraviolet (UV) radiation, with an overall neutral charge. Plasmas can occur in nature and can also be produced artificially by placing a gas under strong electromagnetic fields. Among the different kinds of plasmas that can be artificially generated, CAPs are generated at room temperature and at atmospheric pressure, usually from noble gases (i.e., helium and argon), molecular gases or air directly [34], making them suitable for biomedical applications (Figure 1). Through different physicochemical processes, plasmas lead to environments that are rich in reactive oxygen species (ROS), including the hydroxyl radical (·OH), superoxide anion (O_2_^−^), singlet oxygen (^1^O_2_) and hydrogen peroxide (H_2_O_2_), among others. In addition, reactive nitrogen species (RNS) are also generated, such as nitric oxide (NO), peroxynitrite (ONOO^−^) and other members of the NOx family [35].

CAPs have been demonstrated to produce biological effects such as blood coagulation [36], sterilization [37,38] and wound healing [39,40] due to their ability to generate reactive components that lead to complex biochemical interactions with cells. CAPs have also induced a variety of effects on mammalian cells, ranging from increased cell proliferation to cell death [41], indicating promising clinical uses. An emerging possibility for the clinical application of CAPs is their use to target cancer cells and, therefore, tumor progression. CAPs have shown in vitro efficiency in a large number of cancer cell lines [9] and the ability to reduce tumor size in vivo in animal models [42,43] and a few clinical trials [10,44]. The rich composition of CAPs can induce cytotoxic effects on cancer cells, depending on the time of exposure [45,46,47]. An important advantage of the use of CAP for cancer therapy is that it can affect cancer cells without damaging healthy cells and surrounding tissues, in contrast to conventional chemo- and radiotherapy [48].

### 3.1. Application Methods of Cold Atmospheric Plasma (CAP)

**Figure 2:**
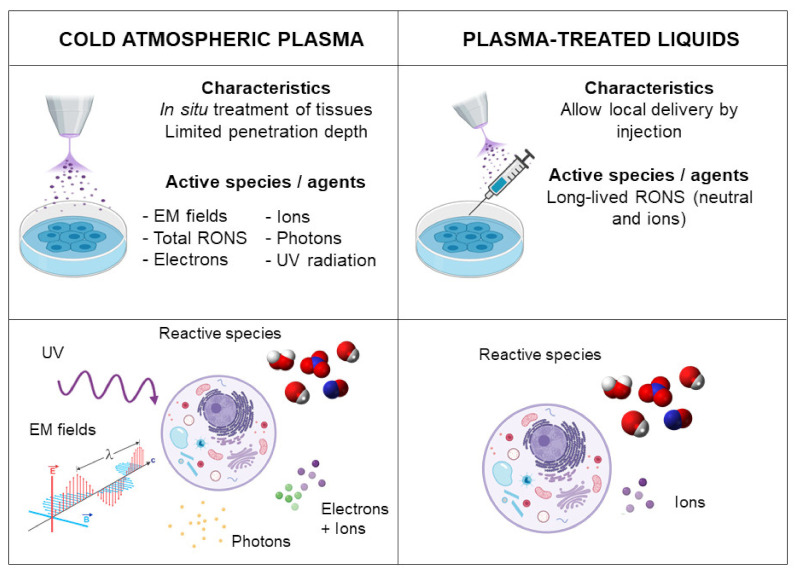
Strategies to apply Cold Atmospheric Plasma (CAP) in cell culture and tissues. In the direct CAP application to cancer cells and tissues, all plasma components (i.e., electromagnetic fields, ultraviolet (UV), total RONS and different particles) can interact with cells, but limitations include the limited depth of penetration. In the indirect treatment, based on plasma-treated liquids, only the most stable reactive species and ions have an effect, while they allow local delivery by injection.

The administration of CAP into the body is an important aspect of the in vivo treatment of tumors. In terms of applicability, two distinct methods are reported in the literature (Figure 2): the direct application to cells or the tumor tissue, where all plasma components are present, and indirect treatment based on the administration of an aqueous solution previously treated by CAP. Both kinds of application have shown efficiency in targeting different types of cancers both in vitro and in vivo [49,50,51,52,53], but there is often a need to increase treatment times in indirect treatments to produce similar cytotoxic effects to the direct treatment [52,53].

On the one hand, in the direct treatment, all plasma components (electromagnetic fields, UV, visible light and short- and long-lived reactive species) act simultaneously on the biological target. For this reason, direct treatment usually induces greater cytotoxicity than indirect treatment as a result of the physical components and short-lived reactive species [52,53]. However, direct CAP has some shortcomings, such as a limited depth of penetration [54], restricting it to superficial types of cancer or requiring direct exposure of the tumor site by open surgery.

In parallel, CAP can be used to generate different reactive species in aqueous-based solutions through diffusion and/or reaction of excited particles from the plasma with the liquid and the transport of RONS generated in the plasma gas phase into the liquid phase [55,56]. The composition and quantity of reactive oxygen and nitrogen species (RONS) are highly dependent on the biochemical composition of the liquid and the plasma parameters employed [56,57,58]. Therefore, these plasma-treated liquids containing more stable long-lived RONS can be applied to cells in vitro or can be locally injected in the tumor in an in vivo situation with a minimally invasive approach, avoiding the need for open surgery.

### 3.2. Anti-Cancer Mechanism of CAP

As previously discussed, CAP contains diverse biochemically active agents. However, the anti-cancer effect of CAP largely depends on the production and the synergistic action of a wide diversity of RONS [59,60,61], along with the additive effects of the physical components (electromagnetic fields and UV in direct treatments). Several RONS are known to play a role in the biological effects of CAP, although H_2_O_2_ and NO_2_^−^ are the most often discussed and quantified for practical reasons [56]. H_2_O_2_ is well-known as an effective inducer of DNA damage and apoptosis in CAP treatment [62], while NO_2_^−^ is a precursor to the intracellular formation of NO, which induces protein and lipid oxidation [63]. The evidence of the synergistic effects of RONS in plasma treatment is that N-acetyl-cysteine (NAC) and carboxy-PTIO, which are H_2_O_2_ and NO scavengers, respectively, are able to inhibit the cell death triggered by CAP treatment [64,65]. Different studies have shown that the H_2_O_2_ and NO_2_^−^ generated by CAP treatment are able to react and produce peroxynitrite (ONOO^−^), which is considered a key mediator of cell membrane peroxidation and increased cell permeability, among other effects [66].

To affect cancer cells, these CAP-generated RONS react with membrane lipids and/or penetrate the cellular membrane, increasing the level of intracellular RONS and triggering oxidative stress [67,68]. This high level of intracellular RONS after exposure to CAP can damage cellular components such as DNA, proteins and lipids. In fact, high expression of PH2A.X, a phosphorylated histone that is used as a DNA damage reporter, has been detected after CAP treatment [60,69]. RONS produced by CAP have been shown to react with amino acids and oxidize lipids present in lipidic bilayers, resulting in cell membrane and organelle damage [70,71,72]. The resulting cellular damage leads to specific signaling cascades that ultimately trigger cell death, mainly by an apoptotic mechanism [73,74,75]. The different CAP-induced signaling pathways are reported to mainly converge in mitochondria, which act as the regulator of apoptosis [64,76,77,78,79,80,81]. As a result of this convergence of different biochemical signals or direct damage induced by CAP-produced RONS, mitochondria increase their transmembrane potential, promoting the release of pro-apoptotic factors that mediate cell death [82,83].

### 3.3. Advantages of Using CAP

The main advantage of using CAP for cancer therapy that has fostered research in this direction is their selectivity, with them having been shown to eliminate cancer cells without damaging healthy cells and surrounding tissues [9]. To explain this, different theories propose cancer cells to be more sensitive to oxidative stress than normal cells due to an elevated metabolic rate, high mitochondrial energetics and alterations of the mitochondrial electron transport chain, all of which lead to the overproduction of intracellular RONS. As a result, a further increase in the concentration of exogenous RONS produced by CAP is thought to overwhelm the tumor cell antioxidant system, leading to oxidative damage, in contrast to what happens in healthy cells, which can manage this increase [84]. It has also been proposed that singlet oxygen produced by CAP inactivates catalase, which is overexpressed on the surface of tumor cells [85], increasing the influx of H_2_O_2_ by aquaporin transporter channels (also overexpressed in cancer) [86], inducing the subsequent depletion of intracellular defenses (i.e., glutathione) and triggering apoptosis [85,87].

The rich composition of RONS produced by CAP also constitutes another advantage over conventional chemo- and radiotherapy, the action of which is also partly based on producing reactive species. Radiotherapy [88] and drugs such as doxorubicin or cisplatin exert part of their effect by increasing intracellular ·OH, so they are limited to its effect and are likely to cause systemic toxicity and side effects [89]. Contrary to these conventional therapies, CAP is applied locally and produces a wide range of RONS that lead to the synergistic effects previously described. Moreover, RNS produced by CAP may also produce specific effects in cancer cells; i.e., NO can disrupt cytochrome C oxidase, resulting in increased levels of ROS, followed by the induction of mitochondrial apoptosis [90].

## 4. Potential Application of CAP in OS

**Figure 3:**
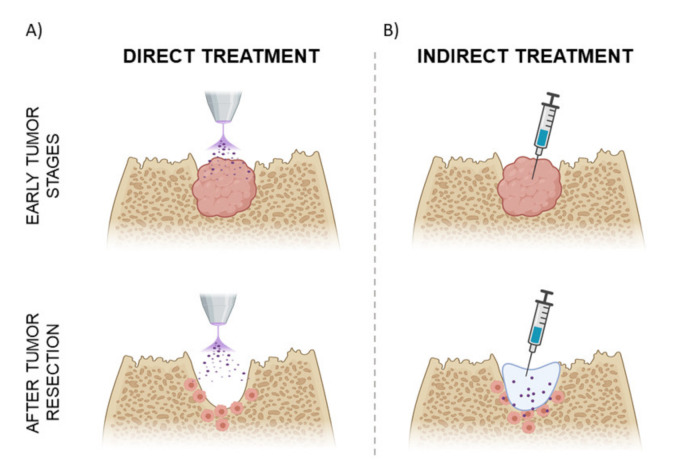
Methods of application of CAP treatment in an OS clinical situation. (**A**) Schematic model of direct treatment of an OS tumor in the early stages of tumor progression or after surgical resection of the tumor. (**B**) Indirect treatment via injection of plasma-treated liquids in the early stages of OS or inside the defect generated after tumor resection.

The two possible approaches to applying CAP, as described in Figure 1, have different implications in terms of their possible translation to clinics for OS treatment. Direct CAP has been proven to reduce the growth of small and localized tumors in in vivo animal models of melanoma and breast cancer [91,92]. However, OS tumors are mostly detected in advanced stages, presenting large volumes [32], which can limit the efficiency of CAP. Moreover, as previously discussed, OS is highly metastatic and may appear disseminated [93]. Considering this, direct treatment may be more indicated in OS at the early stage of localized tumors (Figure 3A) [94]. On the other hand, because a common treatment of OS is a surgical resection of the tumor, direct treatment can also be applied to treat resection margins to eliminate remaining tumor tissue and allow for more conservative surgery (Figure 3A) [54]. The exposure to electromagnetic fields in direct treatment may lead to an increase in cell permeability or cell membrane disruption, which can improve the in situ uptake of RONS [95,96] and drugs [73,97,98]. Moreover, it has been suggested that the destruction of the tumor extracellular matrix (ECM) may improve the response of tumors to chemotherapy or other treatments [99], reducing the effective doses needed for postoperative regimens.

The indirect method allows the tumor site to be reached by injection in a minimally invasive approach and enables repeated doses. The administration of multiple doses of plasma-treated liquids reduced in vivo tumor progression in preclinical models of colorectal and ovarian cancer [100,101,102], allowing for the control of the RONS administered [56,103,104]. Plasma-treated liquids also offer the possibility of being part of a combined or co-adjuvant therapy and have been demonstrated to effectively target different kinds of cancers (i.e., melanoma, ovarian and pancreatic cancer) when combined with drugs [53,102,105], nanoparticles [106] and different cytotoxic molecules [107]. However, plasma-treated liquids may be rapidly washed away from the tumor site, and the presence of ECM antioxidants can cleave the RONS produced, affecting their cytotoxic potential [47,75,104]. To address this scenario, the storage and delivery of plasma-generated RONS in the form of hydrogels is currently under investigation. These biomaterials have been demonstrated to be suitable vehicles for controlled drug release, so this is being approached in CAP therapies by using injectable polymeric hydrogel solutions that are able to cross-link in vivo, opening the door to local and controlled delivery of RONS to the tumor site (Figure 3B) [108,109,110,111].

## 5. In Vitro Effects of CAP in OS

In recent years, an increasing (but still limited) number of studies have produced CAP anti-cancer effects in different OS cell lines by employing direct CAP and plasma-treated liquids (Table 2). In the following sections, we review the two different modes of CAP application in OS cell lines and discuss its potential and the challenges to be faced.

### 5.1. Direct Treatment in OS

**Figure 4:**
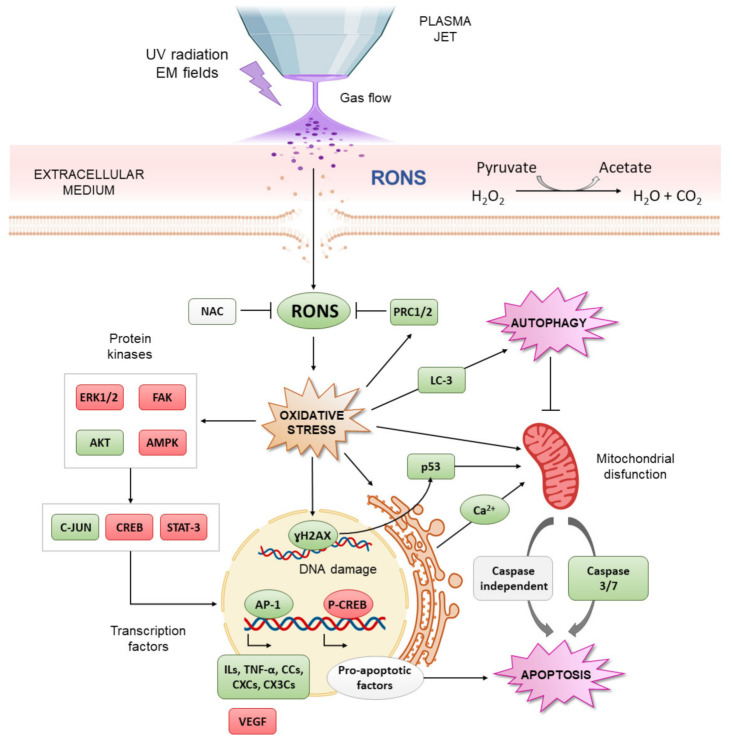
Summary of the main in vitro mechanisms of CAP in OS. Green boxes indicate increase or upregulation, while red boxes indicate decrease or down-expression. CAP containing ultraviolet (UV) and electromagnetic fields produce RONS in liquids. In the cell, pyruvate scavenges hydrogen peroxide. CAP induces cell membrane disruption, which facilitates the transport of RONS into the cell, inducing an increase in intracellular RONS and oxidative stress, which can be attenuated by antioxidants. On the one hand, oxidative stress increases peroxiredoxin expression and modulates protein phosphorylation and transcription factor activation, which determines cell death or survival. CAP-derived RONS can also alter the expression pattern of cytokines, chemokines and growth factors related to OS progression. On the other hand, CAP produces DNA damage that is related to p53 activation and caspase-dependent apoptosis. CAP can also produce mitochondrial injury directly or cause it indirectly by increasing the RE-mitochondrial calcium influx. CAP is also reported to induce mitochondrial autophagy, which may decide between cell death and survival.

Direct application of CAP in OS cells has been reported to produce cell membrane disruption and increase cell permeability, which is considered a key factor in the cellular uptake of CAP-derived RONS [118,119]. In other tumors, this effect has been related to the electrical fields from CAP, which produce similar effects to electroporation [123]. As for other tumors, CAP-generated RONS have been proposed as key mediators of CAP cytotoxicity in OS cells [113,114], as has been confirmed by the alteration of peroxiredoxin expression [116]. Moreover, direct application of CAP is able to produce DNA condensation and fragmentation, followed by activation of p53 [115] and caspase-3/7 [114,115] and then the induction of apoptotic cell death [119] (Figure 4). The selectivity of direct CAP for cancer cells has also been described for OS [104,112,122]: exposure to CAP induces cytotoxic effects, mainly in OS cells, while an increase in cell proliferation with the same treatment is observed in normal bone cells [112]. Conversely, it has also been shown that CAP upregulates different molecules related to OS progression, such as interleukins (ILs), chemokines and growth factors (Figure 4) [117]. In particular, IL-22, which is related to OS invasion [124], is overexpressed, as are chemokines such as CXC, CC, CX3C and C chemokine ligands related to angiogenesis and metastasis in many solid cancers [15], so this aspect deserves careful study. Conversely, vascular endothelial growth factor (VEGF), related to angiogenesis and poor prognosis in OS, is downregulated [125].

### 5.2. Indirect Treatment in OS

Due to the limitations of the direct application of CAP for OS discussed in Section 2.1, indirect treatment has been gaining interest over the last several years, and several works have investigated this topic. Plasma treatments with two main types of liquids have been investigated: cell culture media and saline solutions (Table 2). Canal et al. demonstrated the preferential cytotoxic effect of plasma-treated medium in SaOS-2 cells over healthy bone cells [112]. Mechanistically, plasma-treated medium produces an intracellular ROS increase, DNA damage and apoptosis preferentially in OS cells [104,122] rather than in healthy cells. Suzuki-Karasaki et al. evaluated the ability of plasma-treated medium to induce mitochondrial network aberration and detected autophagy and caspase-independent cell death in different OS cell lines [120,121]. They also reported the induction of cell membrane depolarization and the disruption of Ca^2+^ endoplasmic-mitochondrial homeostasis (Figure 4). These data suggest that, as in other tumor types [126], apoptosis induced by plasma-treated medium can be associated with a mitochondrial pathway in OS (Figure 4).

For the indirect treatment, the CAP parameters employed to produce plasma-treated medium, such as gas flow, distance from the liquid surface, treatment time and the presence of scavengers, such as pyruvate, in the culture medium, have a high impact on the composition of RONS, e.g., H_2_O_2_ production, which has been demonstrated to mediate the selectivity between OS and mesenchymal stem cells (MSCs) [104]. In this sense, a balanced composition of plasma-generated RONS in the medium is key to achieving the desired plasma selectivity.

Tornin et al. demonstrated that exposition of SaOS-2 cells to non-lethal doses of RONS from plasma-treated medium induced the activation of oxidative stress resistance pathways, such as C-JUN and AKT [104], which are involved in OS progression [127,128,129]. Plasma-treated medium also alters cell signaling, which is related to poor prognosis in OS, such as STAT3 [130] and AMPKs [131]. Interestingly, it also induces the downregulation of focal adhesion kinase (FAK), related to invasion and metastasis in OS (Figure 4) [132]. These results show that the cell signaling effects caused by plasma-treated liquids are largely dose-dependent.

In addition, other studies have investigated the cytotoxic potential of saline solutions treated by CAP, which have the advantage of being suitable for clinical application. Cold plasma-treated Ringer’s saline decreased OS cell viability as a function of the concentration of RONS in the liquid phase, and it was shown that high doses of H_2_O_2_ masked the effects of other RONS and led to the loss of anti-cancer selectivity. To date, only one study has investigated the effects of plasma-treated liquids in an ex vivo situation with a real tumor. This work related the RONS delivered in saline solutions to decreased viability in mouse organotypic cultures of OS [122].

## 6. Challenges of CAP for OS Therapy

Currently, less than 5% of the effective treatments against OS tested in vitro have succeeded in clinical trials [133,134,135]. This can be explained by the inability of these in vitro models to recapitulate the in vivo tumor complexity [133]. OS originates in a highly active and rich bone microenvironment, whose composition plays a major role in tumor progression [136]. Moreover, genetic instability and an elevated mutation rate produce a high grade of tumor heterogeneity, which constitutes a key factor in treatment failure [33]. In addition, the prevalence of a tumor subpopulation, namely, OS Cancer Stem Cells (CSCs), has a great impact on drug resistance, tumor progression, relapse and metastasis [137]. These three characteristics explain the poor outcomes in OS treatment (Supplementary Figure S1). For this reason, in the following sections, we discuss how CAP-based therapies could impact these aspects of OS, based on previous research on CAP therapies in other kinds of cancer.

### 6.1. Bone Microenvironment in OS

OS arises in a complex bone microenvironment and interplays with bone cells by releasing extracellular signals in order to promote tumor progression, thereby affecting bone homeostasis [138]. On the one hand, OS is known to disrupt bone remodeling, which is regulated by the balance of osteoblasts, which produce the bone matrix, and osteoclasts, which conduct bone resorption. OS cells disrupt this balance by increasing bone resorption and the release of ECM growth factors, which, in turn, promote tumor cell proliferation [139].

On the other hand, tumor growth and metastasis depend upon the ability to induce nutrient and oxygen supply, so angiogenesis is essential for tumor progression. OS cells respond to conditions such as hypoxia and inflammation by producing stimulating factors, including VEGF, platelet-derived growth factor and endothelin-1 [140]. In parallel, MSCs associated with OS mediate proliferation, metastasis and drug resistance in OS [141,142,143,144] and can acquire a specific cancer-associated fibroblast (CAF) phenotype. Furthermore, MSCs encourage tumor cell growth by secreting a large number of cytokines and growth factors, such as IL-6, TNF-α and IFN-γ; promote angiogenesis by secreting VEGF; and help to evade the immune system by secreting cytokines, such as TGF-β [145,146]. MSCs have also been shown to secrete several pro-inflammatory factors, such as IL-8, and foster OS stemness [147].

OS cells also produce pro-inflammatory factors, which contribute to tumor progression by inducing epigenetic modifications, increasing proliferation and enhancing anti-apoptotic pathways [148]. This deregulation of the inflammatory response is widely associated with immune system evasion by inhibiting the expression of tumor antigens and, therefore, inducing immune tolerance through the secretion of suppressive molecules, such as IL-10, TGF-β and prostaglandin E2, and the expression of inhibitory checkpoint molecules, such as PD-L1 and CTLA-4, and tumor-derived chemokines [149].

How Could CAP Affect the Bone Tumor Microenvironment?

**Figure 5:**
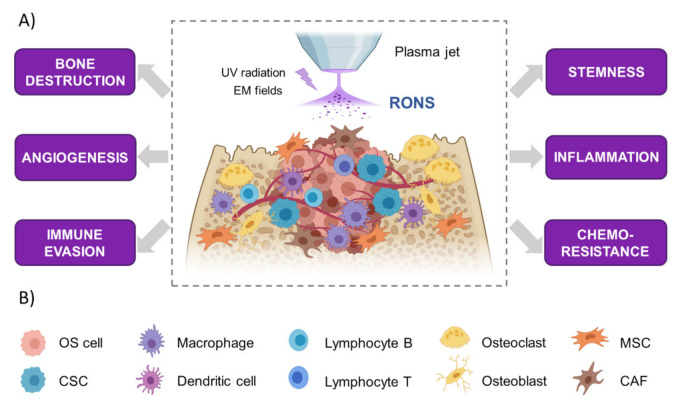
Possible involvement of CAP treatment in the OS microenvironment. (**A**) Schematic illustration of an OS tumor and the main characteristics enhanced by the OS microenvironment that may be affected by CAP. (**B**) Cellular components that are present in the OS microenvironment.

Thus far, no studies have described the effect of CAP on the different components of the OS tumor microenvironment, and this clearly deserves deeper investigation. Diverse results reported in the literature may suggest the possible involvement of CAP in the OS tumor microenvironment (Figure 5A). As described above, the cellular components of the OS tumor microenvironment are mainly composed of osteogenic cells, endothelial cells, MSCs, CAFs and immune system cells (Figure 5B) [136]. CAP is widely described to exert pro-proliferative and pro-differentiative effects in some mesodermal cells [150] and to increase osteoblast differentiation and bone formation [151,152,153]. However, the impacts on bone resorption and osteoclast activity are still unknown. Nanosecond pulsed electromagnetic fields that are produced by several CAPs [154] can induce increases in apoptosis and the OPG/RANKL ratio in OS (key molecules for bone homeostasis), reducing bone destruction [155].

In 2D cultures, treatment with high levels of CAP-derived ROS can affect endothelial cells, which are more sensitive than keratinocytes and fibroblasts, and also reduce tube formation [156]. CAP treatment can also induce cell death in fibroblasts [157], but the effects of CAPs on CAFs have not yet been explored. On the other hand, other studies have shown that plasma-treated media increase the proliferation of MSCs in vitro [104,112]. Given the impact of MSCs on OS progression through the production of metabolites [158] and interleukins [141], the effect of CAP on OS-associated MSCs needs to be deeply understood.

Moreover, CAP treatment increased T cell infiltration in pancreatic cancer, which could be related to the activation of immunogenic cell death in cancer cells [159]. Furthermore, CAP can induce the upregulation of the M1 phenotype in macrophages, presenting increased tumor infiltration ability [100,159]. In vitro studies also suggest that dendritic cells are able to phagocytose pancreatic cancer cells exposed to plasma-treated saline [160]. However, the impact of immune system activation by CAP on OS remains unexplored. Another point that remains to be investigated is the presence of the extracellular matrix, the possible influence of the mineral phase and how it could modify the action of CAP.

### 6.2. Tumor Heterogeneity in OS

OS is one of the cancers with the highest level of heterogeneity in humans. This heterogeneity takes place both at the macroscopic and microscopic levels (at the genomic, transcriptomic and epigenetic levels). Recent investigations have revealed the existence of cancer cells in OS with stemness properties. In this section, we discuss some of the main features of heterogeneity and CSCs in OS and the potential impact of CAP therein.

#### 6.2.1. Oncogenes in OS

OS is characterized by chromosomal instability, which produces a high grade of genetic heterogeneity between patients and within tumor subpopulations, hindering the identification of OS-associated genes. The most common mutations in OS are in the retinoblastoma (p*RB*) and *p53* tumor suppressor genes. The wild-type p53 protein regulates genes involved in DNA repair, cell cycle checkpoints and apoptosis initiators [161], and its inactivation has been identified as an initiating event in OS onset [162]. On the other hand, pRB is reported to promote osteogenic differentiation, and its mutations could act synergistically with *p53* inactivation in OS formation [163].

Other types of proto-oncogenes associated with OS are protein kinases such as MAPKs and PI3K/AKT/mTOR pathways [130]. These biochemical cascades integrate signaling inputs from the tumor microenvironment and direct the activity of different effector proteins. MAPKs can constitutively activate several transcription factors, such as C-MYC and C-FOS, which control different cell functions that promote cancer progression [164]. On the other hand, PI3K/AKT/mTOR can inactivate pro-apoptotic factors such as Bad and Procaspase-9, increasing cell survival, and drive the expression of pro-angiogenic factors [165].

Moreover, the previously mentioned transcription factors C-FOS, C-JUN and C-MYC are commonly found to be overexpressed in OS [127,166]. These transcription factors, activated from upstream pathways, bind to specific sites of DNA and promote the expression of several genes involved in different cancer hallmarks, including cell proliferation, differentiation and survival [167,168,169].

#### 6.2.2. How Could CAP Affect Intracellular Signaling in OS?

Little is known about the effect of CAP in OS intracellular signaling, so the results observed in other tumor types are discussed here as potential indicators of the OS cell response. In both direct and indirect CAP-based therapies, activation of p53 is reported to be the main mediator of cell apoptosis in different kinds of cancers [74,82,170,171]. Activating p53 is an attractive approach for blocking tumor progression. In the case of OS, different mutations or delection in p53 are reported in approximately 50% of patients [172]. This variability is reflected in the different p53 status found in the different cell lines derived from OS (SaOS-2, MG-63, U2-OS, etc.) [173], which might lead to heterogeneous responses to CAP. In this sense, the consequences of activating mutant p53 by CAP in OS are not completely clear and need further investigation.

Furthermore, MAPK signaling plays a crucial role in OS, and the effects of CAP treatment are still poorly understood. In other kinds of cancers (i.e., glioblastoma, head and neck, melanoma and ovarian cancers), the activation of p38 and downregulation of ERK are widely reported to be highly related to apoptosis induced by CAP [46,76,77,174,175,176,177], whereas healthy cells increase ERK to promote cell proliferation. In the case of OS, in SaOS-2 cells exposed to plasma-treated medium with low cytotoxicity, phospho-ERK1/2 activation was coupled to cell proliferation, while highly cytotoxic plasma-treated medium downregulated ERK [104], and in both cases, p38 phosphorylation was not detected. These data seem to indicate that the p38/ERK pathway is not related to apoptosis induced by CAP in OS. Moreover, plasma-treated medium induces the activation of C-JUN, which is related to pro-tumoral signaling [104]. In this sense, the involvement of CAP treatment in MAPK signaling in OS must be more deeply investigated. In sum, there is not yet enough evidence to unravel the molecular mechanisms associated with the oxidative stress induced by CAP in OS cancer cells, as the few reports related to cell signaling by CAP in other kinds of cancers propose the same cell signaling as that affected by the classical oxidative stress therapies [43,46,177,178,179].

#### 6.2.3. Cancer Stem Cells in OS

It is well documented that mutated MSC-derived osteogenic progenitors or undifferentiated MSCs under the influence of normal bone microenvironment signals act as the cells of origin in OS [180]. Experimental evidence shows that OSs are sustained by subpopulations of self-renewing cells that can generate the full phenotypes of tumor cells [137]. This subpopulation is known as CSCs, which are tumor-quiescent and possess stem properties such as self-renewal, pluripotency and cell differentiation into mesenchymal lineages. Their characteristics include upregulated embryonic genes and drug transporters, high aldehyde dehydrogenase (ALDH) activity and higher DNA repair capacities [181]. These characteristics make these cells highly resistant to conventional therapies and capable of differentiating and reforming OS tumors, and they are proposed to be responsible for tumor relapse after treatment cessation [137].

Moreover, CSCs are suggested to contain lower ROS levels than their corresponding non-tumorigenic cells to maintain their stem properties, which can be associated with high expression of ROS-scavenging molecules [182,183]. In addition, CSCs can adapt to oxidative stress by altering their metabolic profile, switching between OXPHOS, glycolysis and pentose phosphate pathways [182]. Therefore, to develop new effective therapies against OS, the effect on targeting CSCs must be evaluated.

#### 6.2.4. How Could CAP-Induced Oxidative Stress Affect CSCs in OS?

Currently, there are no studies evaluating the effect of CAP in OS-CSCs, and only a couple of studies have described the impact of CAP on cancer-initiating cells in the case of endometrial carcinoma [184,185]. In this kind of cancer, Ikeda et al. showed that CAP reduced high-expressing ALDH cells using both direct treatment and a plasma-activated medium. On the other hand, there are no studies reporting how CAP treatment affects CSC metabolism. However, a few works have addressed the effect of CAP on mitochondrial metabolism, showing a decrease in glycolysis in glioblastoma [186] or OXPHOS in melanoma [80] and skin cells [187], which may elucidate a possible effect of CAP on CSC metabolism.

Despite these promising results, the effects of CAP could be completely different for the case of OS-CSCs. As previously mentioned, an increasing number of studies suggest that CSCs are more resistant to RONS than normal cancer cells, which is related to their antioxidant ability and their metabolic status [182,188]. The metabolic status of CSCs is controversial and can be tumor or cell line-dependent. OS-CSCs rely on OXPHOS, with the potential to switch to glycolysis depending on microenvironmental requirements [189]. In an in vitro study, the OS-CSC phenotype had a higher glycolytic rate than the parental OS, and OXPHOS was inhibited by treatment with cisplatin [190]. In this study, the inhibition of PKM2, a glycolytic protein, increased the sensitivity of OS-CSC to cisplatin treatment [190]. Considering these data, there is an urgent need to study how the oxidative stress produced by CAP can affect the metabolic reprogramming capacity and the stemness properties of OS-CSC.

## 7. Future Trends

CAPs have been demonstrated to selectively induce anti-cancer effects in different OS cell lines in vitro using both direct CAP and plasma-treated liquids [104,112,113,114,115,116,117,118,119,120,121,122]. Although these promising data provide relevant information for understanding CAP applications in OS, several key aspects of OS progression have not yet been considered.

For instance, the repercussions of patient heterogeneity, tumor subpopulations and tumor microenvironment components need to be evaluated.

The selectivity of CAP is based on the differential levels of RONS between cancer and normal cells, which makes cancer cells more sensitive to pro-oxidant induction by the treatment [50]. However, this model does not consider the different vulnerabilities of OS subpopulations to RONS, i.e., CSCs, CAFs or MSCs, and deserves careful investigation.

The cell signaling affected by CAP in OS is still poorly studied, and their possible effects on OS progression [191] and the CSC phenotype [192] need to be deeply understood. The previously mentioned targeted strategies against these signaling molecules have also been demonstrated to effectively target the CSC subpopulation that can resist oxidative stress. In OS, different small molecules [179,193], si-RNA [29,30,194,195] and CRISPR-Cas technologies have been proposed to target several genes, such as mTOR and C-MYC. As described by Tornin et al., heat shock protein (HSPs) expression is closely related to CAP therapy [104], making it an interesting molecule for targeted treatments. Inhibition of HSPs presents great potential for OS therapies [196,197,198]. Moreover, the combination of CAP treatment and inhibition of HSP90, another member of the HSP family, has been demonstrated to be effective in several cancer cell lines [199].

As reflected in Table 2, almost all studies on CAP effects on OS have been performed in adherent or floating cell cultures. It has been amply demonstrated that cells cultured in 2D are not representative of the cells present in tumors because they lack cell-to-ECM interactions [134]. In contrast, 3D culture models provide a more complex scenario that mimics the interactions between cells and ECM by employing culture systems that induce the production of ECM (i.e., spheroid and organoid cultures) or using scaffolds composed of several ECM components, including, in this case, the mineral matrix. In these culture models, OS cells present more similar protein expression profiles, metabolism, signal transduction, mechanical properties and responses to stimuli to those in in vivo treatment [134], offering a more realistic tool to approximate the in vivo situation with CAP in vitro results.

Investigating the effect of CAP in OS co-cultures with OS-associated cells is also required to evaluate the impact of CAP-based therapies on processes such as angiogenesis, immune response and stroma interaction and bone remodeling [134,200,201,202,203]. Of course, after suitable validation in OS in vitro 3D models, CAP-based therapies will have to be evaluated in the preclinical in vivo scenario.

## Figures and Tables

**Table 1:** Current strategies for osteosarcoma (OS) treatment: Summary of the main mechanisms of action, benefits and disadvantages.

	BASIS	ADVANTAGES	DISADVANTAGES
**SURGERY**	Limb amputation; Surgical resection of tumor tissue	↑ Tumor remission and survival in non-metastatic OS patients.	↑ Tumor residues, relapse and limb disfunction. ↓ Effectiveness in metastatic OS patients.
**CHEMO-** **THERAPY**	Methotrexate, doxorubicin and cisplatin: inhibits DNA synthesis; Doxorubicin and cisplatin: Free radical production	↓ Tumor growth; Tumor remission facilitates surgical resection; Eradicates tumor remnants and distal metastasis.	Drug resistance in many patients; Crystal nephropathy; Systemic oxidative stress: hepatotoxicity and cardiotoxicity; Hepatotoxicity, cardiotoxicity, altered bone remodeling function, side effects.any side effects.
**RADIOTHERAPY**	Radiation-induced DNA damage; Production of hydroxyl radicals (·OH).	To control of resection margins; Local control of OS tumors that cannot be properly resected.	↓ Response of OS tumors and a need for ↑ doses; Detrimental effect on normal tissue; Systemic oxidative stress and cytotoxicity; Risk of a radiation-induced secondary tumor.
**IMMUNOTHERAPY**	Use of components of the immune system to increase the immune response against cancer cells.	↓ Side effects than chemo- and radiotherapies and risk of tumor relapse.	↑ Capabilities of OS to ignore immune system; Autoimmune responses.
**TARGETED THERAPIES**	Use of different kinds of inhibitors of critical proto-oncogenes.	Targeted for OS cells; Free of systemic effects.	↑ Difficulty to identify relevant proto-oncogenes in OS.

**Table 2:** Summary of in vitro studies on CAP applications for OS treatment.

Cell Lines	CAP Device	Cell Response	Refs
**DIRECT CAP TREATMENT** **→ floating cultures except ^α^**			
SaOS-2, hMSCs hOBs ^α^	He APPJ *	Cytotoxicity of cancer cells to CAP rather than healthy bone cells.	[112]
U2-OS, 3T3	Maxium^®^ CAP Coagulator 1000 kINPen MED	Differential ↓ in proliferation depending on the plasma jet.	[113]
U2-OS, MNNG/HOS	kINPen MED MiniJet-R	Plasma jet-dependent response; ↓ cell proliferation; activation of caspase-3/7.	[114]
	kINPen MED	↓ Cell proliferation; p53 phosphorylation; DNA condensation and nuclear degradation.	[115]
		↓ Cell proliferation and peroxiredoxin expression; NAC-mediated reduction of CAP cytotoxicity.	[116]
		Cell line-dependent chemokine and cytokine modulation.	[117]
		↑ Cell membrane permeability.	[118]
		↓ Cell proliferation and cell membrane permeability; apoptotic cell death.	[119]
**INDIRECT CAP TREATMENT (PLASMA-TREATED LIQUIDS) adherent cultures except ^β^**			
HOS, SaOS-2, 143B	DBD *	Mitochondrial network aberration, ↑ autophagy.	[120]
HOS, SaOS-2, 143B, hFOB, LM8, K7M3, MC-3T3		Cytotoxic effect in transformed cells; mitochondrial network aberration; caspase-independent cell death; cell membrane depolarization; Ca^2+^ homeostasis disruption.	[121]
SaOS-2, hMSCs, hOBs	He APPJ	↑ Cytotoxicity of CAP to cancer cells than healthy bone cells and apoptosis; ↓ focal adhesions.	[112]
SaOS-2, hBM-MSCs		Selective cytotoxic effects depending on H_2_O_2_ generated and the presence of pyruvate, ↑ DNA damage and apoptosis, phospho-kinase alterations.	[104]
SaOS-2, MG-63, U2-OS, hBM-MSCs	He APPJ kINPen IND	Selective cell death depending on plasma jet and RONS concentration, induction of intracellular ROS increase, DNA damage and apoptosis between healthy and cancer cells.	[122]
Tumors produced from MOS-J ^β^	He APPJ	↓ Proliferating cells and viability.	[122]

* APPJ: Atmospheric Pressure Plasma Jet; DBD: Dielectric Barrier Discharge. **^α^** Adherent cultures. **^β^** Floating tumor tissues.

## Data Availability

No new data were created or analyzed in this study. Data sharing is not applicable to this article.

## Supplementary Materials

The following are available online at https://www.mdpi.com/2077-0383/10/4/893/s1, Figure S1: Main characteristics of OS tumors which may have great impact in CAP-based therapies. Briefly, (1) the interaction with bone microenvironment, (2) the different characteristics between OS subtypes and (3) the presence of different phenotype within OS tumors and the presence of Cancer Stem Cells (CSC) can lead to different response to CAP-based therapies that have to be evaluated. MSCs: Mesenchymal stem cells; CAFs: Cancer-Associated Fibroblasts.

## Abbreviations

ALDH	Aldehyde dehydrogenase
CAP	Cold atmospheric plasma
CAF	Cancer associated fibroblast
CSC	Cancer stem cell
ECM	Extra-cellular matrix
HSP	Heat-shock protein
IL	Interleukin
MSC	Mesenchymal stem cell
OS	Osteosarcoma
pRB	Retinoblastoma
RNS	Reactive nitrogen species
RONS	Reactive oxygen and nitrogen species
ROS	Reactive oxygen species
UV	Ultraviolet
VEGF	Vascular endothelial growth factor
