# Supplementary material for: Cold Atmospheric Plasma: A New Strategy Based Primarily on Oxidative Stress for Osteosarcoma Therapy
Source: J Clin Med. 2021 Feb 23;10(4):893. doi: 10.3390/jcm10040893 (PMC7926371; doi:10.3390/jcm10040893)
Supplement: Supplementary file 1 [file jcm-10-00893-s001.pdf]

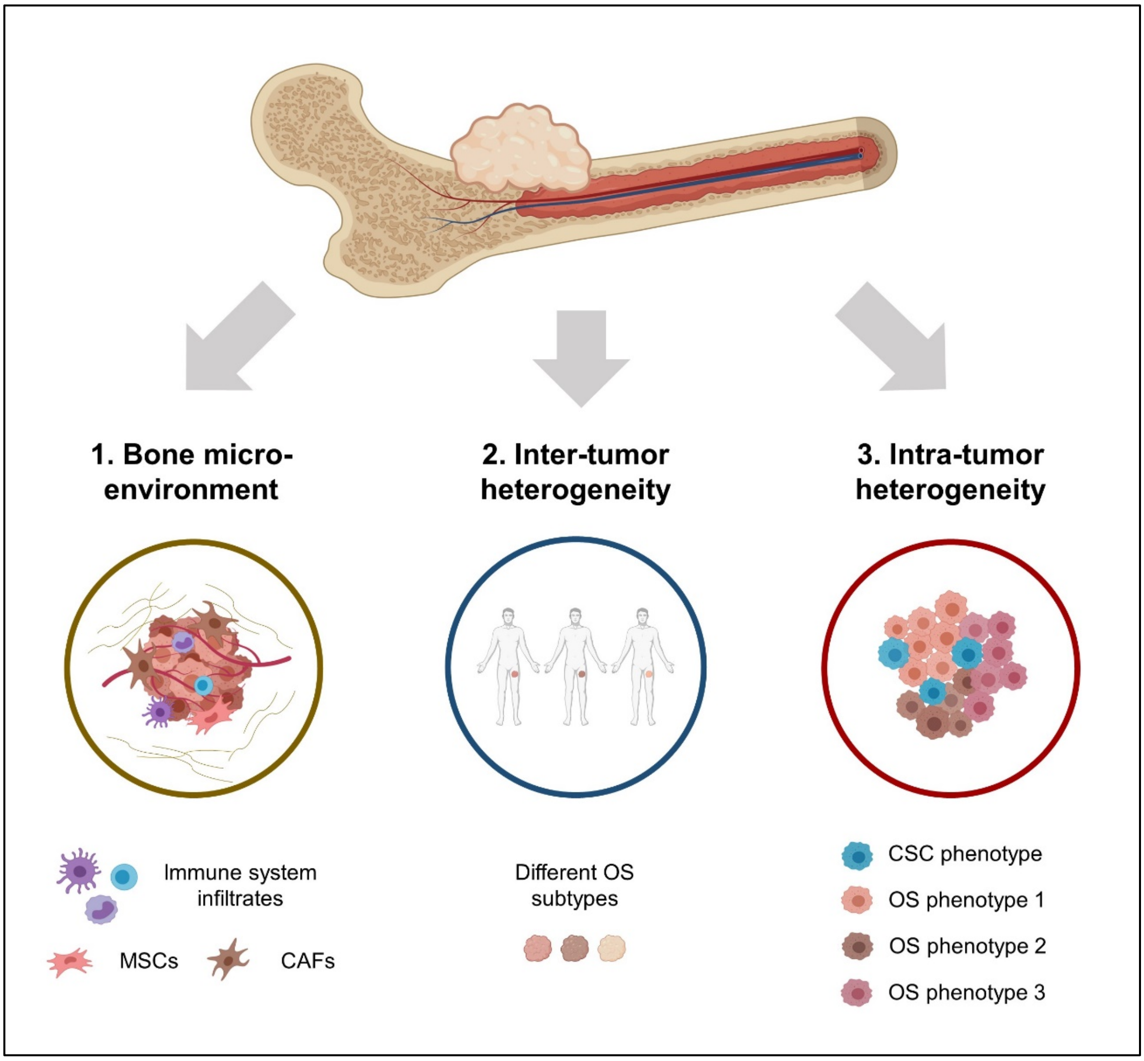

FIGURE S1. Main characteristics of OS tumors which may have great impact in CAP-based therapies. Briefly, (1) the interaction with bone microenvironment, (2) the different characteristics between OS subtypes and (3) the presence of different phenotype within OS tumors and the presence of Cancer Stem Cells (CSC) can lead to different response to CAP-based therapies that have to be evaluated. MSCs: Mesenchymal stem cells; CAFs: Cancer-Associated Fibroblasts.
